# Employing General-Purpose and Biomedical Large Language Models with Advanced Prompt Engineering for Pharmacoepidemiologic Study Design

**DOI:** 10.1007/s11095-026-04190-5

**Published:** 2026-09-11

**Authors:** Xinyao Zhang, Nicole Sonne Heckmann, Manuela Del Castillo Suero, Francesco Paolo Speca, Maurizio Sessa

**Affiliations:** Department of Drug Design and Pharmacology, University of Copenhagen, Jagtvej 160, 2100 Copenhagen, Denmark

**Keywords:** artificial intelligence, large language model, pharmacoepidemiology, real-world evidence

## Abstract

**Background:**

The potential of large language models (LLMs) to automate and support pharmacoepidemiologic study design is an emerging area of interest, yet their reliability remains insufficiently characterized. General-purpose LLMs often display inaccuracies, while the comparative performance of specialized biomedical LLMs in this domain remains unknown.

**Methods:**

This study evaluated general-purpose LLMs (GPT-4o and DeepSeek-R1) *versus* biomedically fine-tuned LLMs (QuantFactory/Bio-Medical-Llama-3-8B-GGUF and Irathernotsay/qwen2-1.5B-medical_qa-Finetune) using 46 protocols (2018–2024) from the HMA-EMA Catalogue and Sentinel System. Performance was assessed across relevance, logic of justification, and ontology-code agreement across multiple coding systems using Least-to-Most (LTM) and Active Prompting strategies.

**Results:**

GPT-4o and DeepSeek-R1 paired with LTM prompting achieved the highest relevance and logic of justification scores, with GPT-4o-LTM reaching a median relevance score of 4 in 8 of 9 questions for HMA-EMA protocols. Biomedical LLMs showed lower relevance overall and frequently generated insufficient justification. All LLMs demonstrated limited proficiency in ontology-code mapping.

**Conclusion:**

Off-the-shelf general-purpose LLMs currently offered more reliable support for pharmacoepidemiologic study design than the smaller biomedical LLMs evaluated. In the model-controlled comparison (GPT-4o with Least-to-Most *versus* Active prompting), prompt strategy did not significantly affect performance (paired *p* = 0.93), and prompt comparisons are therefore reported descriptively. Because the two model groups also differed in scale and instruction-tuning, this contrast should be interpreted as a comparison of readily deployable options rather than of biomedical specialization alone.

**Supplementary Information:**

The online version contains supplementary material available at https://doi.org/10.1007/s11095-026-04190-5.

## Introduction

The growing need for rapid evidence generation in pharmacoepidemiology places strong demands on study design and data quality [1]. This is particularly important for populations underrepresented in clinical trials and comparative effectiveness/safety research in routine care. [2, 3] As artificial intelligence (AI) becomes increasingly integrated into biomedical research, large language models (LLMs) have been proposed as tools to support time- and resource-consuming tasks, including those relevant to pharmacoepidemiology. [4–6] Interest in this area is growing within regulatory settings. The U.S. Food and Drug Administration (FDA) has explored the use of LLMs such as BERTox and GPT to support document analysis, literature review, and toxicity assessment. [5] The European Medicines Agency (EMA) has issued high-level recommendations on the use of LLMs in regulatory activities. [6] Despite this momentum, the role of LLMs in time- and consuming-tasks in pharmacoepidemiology, such as study design, remains unclear.

Pharmacoepidemiologic study design requires accurate identification and formulation of multiple interrelated methodological elements, including temporal assessment windows, inclusion and exclusion criteria, exposure definition, and ontology-code mapping. [7–9] Early attempts to use off-the-shelf LLMs for these tasks have highlighted limitations. [10–12] However, it remains uncertain whether such limitations are mainly related to the type of model (i.e., general-purpose), prompt strategy, or both. This study therefore aimed to evaluate the performance of off-the-shelf general-purpose LLMs and biomedical LLMs, combined with prompt-engineering strategies, for supporting pharmacoepidemiologic study design.

## Methods

### Data Sources

This study evaluated LLM performance using study protocols from two pharmacoepidemiologic sources (i.e., HMA-EMA Catalogue and Sentinel System). From the HMA-EMA Catalogue of Real-World Data (RWD) Studies, all available DARWIN EU® protocols were included at the time of analysis (*n* = 16). In addition, 15 non-DARWIN expert-developed protocols were randomly sampled from the HMA-EMA Catalogue and were required to be registered between January 1 st, 2023, and February 22nd, 2024, finalized, written in English, and publicly available. From the Sentinel System, 15 protocols were selected registered between January 1 st, 2022, and February 22nd, 2024, marked as complete, classified as “drug and outcome analysis” or “drug use,” and publicly available. [13, 14]

### Pharmacoepidemiologic Design Concepts

The evaluation framework was structured around key pharmacoepidemiologic design concepts derived from the HARPER checklist. [15] These concepts were operationalized as a sequence of concept-specific tasks to reflect the main elements typically required for protocol design in RWD studies. The concept-specific tasks were structured as follows: a) Question 1 assessed the correct identification of the study design; b) Question 2 assessed the definition of the index date; c) Question 3 assessed inclusion and exclusion criteria; d) Question 4 assessed the inclusion and exclusion assessment window; e) Question 5 assessed exposure; f) Question 6 assessed the outcome; g) Question 7 assessed the follow-up period; h) Question 8 assessed covariates; i) Question 9 assessed the covariate assessment window; and then it was separately assessed ontology-code mapping.

### Human Expert Gold Standard

From each protocol, 2 human assessors (XZ and MS) extracted pharmacoepidemiologic design concept mentioned in Sect. "Pharmacoepidemiologic Design Concepts", which served as the gold standard against which LLM outputs were evaluated. Specifically, they included study design, index date, inclusion and exclusion criteria, inclusion and exclusion assessment window, exposure, outcome, follow-up period, covariates, covariate assessment window, and ontology-code mapping. [15] LLM-generated outputs were compared with protocol-derived expert answers across the 10 questions (Table 1 provides the specific prompts used for each question). The extraction targeted information explicitly stated in each protocol, making it an objective transcription task rather than a subjective judgement. The two assessors (XZ and MS) extracted these elements independently; the few discrepancies that arose were typographical or concerned the location of the information within the document and were resolved by consensus before any comparison with LLM outputs. The complete gold standard, all protocols, and all LLM outputs are publicly available in the study repository, allowing independent verification against the source protocols. LLM outputs were subsequently rated against this fixed, pre-specified gold standard, and scoring disagreements were resolved by discussion. Relevance and reasoning-concordance outcomes were assigned by GPT-4o acting as a single automated evaluator; the protocol-derived reference set and the human logic of justification were produced by two pharmacoepidemiologists (MS and XZ), who discussed all items and reached consensus in plenary discussion, yielding a single agreed rating per item; a formal independent inter-rater reliability was therefore not computed. Ontology-code mapping used rule-based matching against the protocol-derived reference set.

**Table 1:** Prompt Examples

Concept	Least to Most	Active prompt
Study design	Based on the title [TITLE], list all the candidate study design types (e.g., cohort study, case–control study) and select the most appropriate one and justify it	Primary prompt: As a pharmacoepidemiologist, recommend the optimal study design type for the study titled "{TITLE}". Consider all candidate designs (e.g., cohort, RCT, case control). Constraints: 1. Align with CONSORT/STROBE guidelines 2. Prioritize feasibility in real-world settings 3. Explicitly state rejection reasons for alternative designs Then enhance prompts:
		[CoT Example]
		Objective: Assess long-term effects of Drug X on risk Step 1: Identify need for longitudinal exposure-outcome data → Cohort design Step 2: Check feasibility of randomization → Reject RCT (lack of equipoise) Step 3: Compare with case–control → Prefer cohort for temporal clarity
Index date	Define the index date for this study based on the selected [Answer to Question 1] study design, indicating its specific definition and whether it is fixed or variable	Primary prompt: Define index dates of this study. Requirements: 1. For pharmacological studies: Use first prescription date + 30-day washout 2. For surgical studies: Use procedure date ± 7-day pre-op assessment 3. Flag potential immortal time bias sources
		[Validation] Check FDA Sentinel Common Data Model, DARWIN, HMA-EMA for alignment
Inclusion and exclusion criteria	Inclusion criteria (e.g., age ≥ 18 years, confirmed disease) and exclusion criteria (e.g., past medical history, missing data) should be developed for this study based on the [Q1 answer] design and [Q2 answer] index date	Generate inclusion/exclusion criteria of this study. Mandatory elements: 1. Explicit linkage to objective-specific endpoints 2. Use WHO International Classification of Functioning (ICF) framework 3. Flag criteria causing selection bias > 20%
Inclusion and exclusion assessment window	Based on the [answer to question 2] index date, identify the time window for assessing inclusion and exclusion criteria (e.g., a baseline period of 6 months before the index date, an exclusion period of 1 year after the index date) and its specific duration	Primary prompt: Define temporal assessment windows for inclusion/exclusion criteria of the answer to question 3
		Constraints: 1. Align with EMA Guideline on GCP (Rev 3) 2. Use moving window approach for chronic conditions 3. Specify allowable overlaps (± 5% timeline tolerance) Enhance prompt:
		[CoT Example]
		Objective: Window Logic: 1. Baseline: 2. Exclusion:
		[Check] Apply inverse probability weighting for missing window data
Exposure	Based on the [Answer to Question 1] design and [Answer to Question 3] inclusion criteria, define exposure (e.g., drug dose, exposure duration)	Primary prompt: Define study exposure of the study Required elements: 1. Dose–response granularity (ATC + RxNorm mapping) 2. Exposure lag periods with biological plausibility check
		Competing risk adjustment plan
Outcome	Based on the [answer to question 1] design, specify primary/secondary outcome definitions (e.g., laboratory confirmed diagnosis, imaging evidence)	Primary prompt: Specify outcome for this study. Must include: 1. Primary/secondary endpoints 2. Competing risk definitions (e.g., death censoring rules)
		Sensitivity analysis protocols for outcome misclassification
Follow-up period	In conjunction with [Q.2 answer] Index date and [Q.6 answer] Definition of ending, set the start and end of the follow up period, whether right censoring is allowed and the minimum length of follow-up (e.g. 1 year)	Question 7: Primary prompt: Define follow-up periods in this study Requirements: 1. Account for disease-specific latency periods 2. Apply landmark analysis for time-varying exposures 3. Specify left-truncation handling methods Enhance prompt:
		[CoT Example]
		Objective: Follow-up Logic: 1. Start: 2. End:
		Censor: Death
Covariate	List covariates (e.g., age, sex, comorbidities, medication history) to be adjusted for based on [QUESTION 5 ANSWER] exposure and [QUESTION 6 ANSWER] outcome	Primary prompt: Identify covariates in this study Must: 1. Categorize into confounders/mediators/effect modifiers 2. Provide LOINC codes for lab covariates 3. Include negative control variables
		Enhance prompt: [CoT Example]
		Objective:
		Covariates: 1. Confounder: 2. Effect modifier: Negative control:
Covariate assessment window	Based on the [QUESTION 2 ANSWER] index date, define the time window in which the covariates will be evaluated (e.g., laboratory data within 1 year prior to the index date)	Primary prompt: Define temporal windows for covariate assessment in this study. Constraints: 1. Align with FDA’s Structured Product Labeling (SPL) standards 2. Handle time-varying covariates using marginal structural models 3. Specify missing data imputation thresholds Enhance prompt:
		[CoT Example]
		Objective:
		Window Strategy: • Static: • Time-varying: Lagged:
Ontology-code	Convert diagnoses, exposures, outcomes from [Q5/6 answers] to standard codes i.e. ICD-10, ATC	Map all medical concepts in previous answers to standard terminologies based on the answer to question 5, 6 list all the code of this concept. Required systems:

### Prioritization of Large Language Models and Prompt Strategies

Two general-purpose LLMs (GPT-4o—snapshot gpt-4o-2024–08-06 and DeepSeek-R1) and four biomedical LLMs were included in the screening phase: QuantFactory/Bio-Medical-Llama-3-8B-GGUF, mradermacher/DeepSeek-r1-Medical-Mini-GGUF, Plaban81/gemma-medical_qa-Finetune, and Irathernotsay/qwen2-1.5B-medical_qa-Finetune. [16–19] Model selection for biomedical LLM was based on overall performance as reported by the Open LLM Leaderboard hosted on Hugging Face (https://huggingface.co/spaces/open-llm-leaderboard/open_llm_leaderboard#/) as of March 2025. LLMs were paired with seven prompt engineering strategies identified from the literature: 1) basic prompt with term definitions, 2) synthetic prompting, 3) active-prompt, 4) plan-and-solve, 5) least-to-most (LTM), 6) Tree-of-Thought, and 7) decomposition prompting. [20–22] Together, this yielded 42 model-prompt combinations for the initial pre-assessment of the most performing combinations. Prompt construction was guided by the CLEAR framework, which emphasizes concise, logical, explicit, adaptive, and reflective prompt design. [23] A pre-assessment was conducted on three randomly selected protocols, one from each protocol source. Model-prompt combinations were evaluated against the human expert gold standard using a 1–5 Likert scale for relevance and logic of justification, a categorical agree/partly/disagree measure for GPT-4o-assessed reasoning concordance, and error frequency as an additional criterion. Based on this screening, the five top-performing combinations were selected for the main analysis: GPT-4o with LTM, GPT-4o with Active Prompt, DeepSeek-R1 with LTM, QuantFactory/Bio-Medical-Llama-3-8B-GGUF with LTM, and Irathernotsay/qwen2-1.5B-medical_qa-Finetune with LTM. Protocols, extracted data, and LLM-generated output from the pre-assessment are available at: https://github.com/madelsu/LLM-for-pharmacoepi-study-design/tree/main/Data/pre_assessment. The pre-assessment was a feasibility-driven prioritization rather than an exhaustive comparison: combinations were eliminated primarily on the basis of gross, categorical failures (e.g., model crashes, prompt echoing, large volumes of irrelevant auto-generated questions, or failure to follow the intended algorithm) that were severe and stable rather than marginal, and the five carried-forward combinations deliberately retained both general-purpose and biomedical models and the two best-performing prompt strategies. Because screening was conducted on three protocols only, the complete pre-assessment data are released so that the basis for each exclusion can be independently audited. Specifically, model–prompt configurations were prioritized based on performance in a three-protocol feasibility screen of 42 combinations (Appendix 1). Among the seven prompting strategies, Least-to-Most prompting was the most consistent across model families, it had the highest mean screening score across families and ranked first or second in every family, whereas zero-shot prompting was competitive only for GPT-4o and ranked near-lowest for the smaller models, and was therefore adopted as the common advanced-prompting strategy for the cross-model comparison. Active Prompting was additionally retained for GPT-4o to enable the model-controlled prompt comparison; zero-shot, the baseline, was not carried forward as the study targets advanced prompt engineering, and its GPT-4o screen score was within 0.04 of the retained arms. PMC-LLAMA had the highest Least-to-Most screening score, and for it Plan-and-Solve scored marginally higher than Least-to-Most (3.70 *versus* 3.59), but Least-to-Most was adopted as the common cross-model prompt. Qwen2-1.5B-medical was retained at its best-scoring prompt (Least-to-Most). DeepSeek-R1 was included *a priori* as the second leading general-purpose reasoning comparator and was run under the common Least-to-Most prompt; it was not part of the numeric prompt screen, so its screening ratings are unpopulated.

### Study Outcomes

The primary outcome was relevance, defined as agreement between LLM-generated answers and protocol-derived expert answers measured with Likert-scale. Agreement was considered acceptable if scores were identical or differed by no more than one Likert level. Logic of justification was assessed as a key secondary outcome using a 5-point scale, with 1 indicating illogical or inconsistent reasoning and 5 indicating coherent, well-supported, and methodologically rigorous reasoning. Ontology-code agreement was assessed by comparing predicted and reference codes (i.e., from the protocols) for exposure and outcome across ontology groups and summarizing percentage agreement by model, prompt strategy, and protocol source. Error patterns were examined through qualitative review of predefined error types, and frequencies were summarized descriptively.

### Data Analysis and Software

A descriptive analysis of the included protocols was performed by protocol source (DARWIN EU®, HMA-EMA, and Sentinel System). Counts and proportions were calculated for publication year, geographical origin of authors, study type, database use, and ontology-code mappings used to define exposure and outcome. For relevance and logic of justification, Likert-scale results (1–5) were summarized for each LLM, prompt strategy, and protocol source using medians, interquartile ranges (IQRs), and means, and each comparison was rendered with a distinct, fit-for-purpose visualization. Relevance was displayed by LLM as a diverging stacked bar chart of the full Likert distribution, by prompt strategy as a bubble plot of mean scores across pharmacoepidemiologic concepts, and by protocol source as a percentage histogram contrasting biomedical and general-purpose (non-biomedical) models. Logic of justification was displayed by LLM as an annotated heatmap of mean scores across concepts, by protocol source as a Cleveland dot (lollipop) plot of means with standard-error bars and by prompt strategy as a violin plot. In all figures, color distinguished biomedical from general-purpose models. Ontology-code agreement was calculated as percentage agreement between model-predicted and reference codes and was summarized by ontology-code group, model, prompt strategy, and protocol source. Results were visualized using comparative bar plots. In addition, set-based agreement metrics (precision, recall, F1, Jaccard, and exact-set agreement) were computed per configuration and coding system, and every non-matching output was classified into a predefined error taxonomy; native SNOMED CT and RxNorm identifiers evaluated against OMOP standard concept IDs were reported separately as coding-convention differences rather than errors. Error frequencies were tabulated and summarized descriptively. To explore error patterns across questions and biomedical LLMs, deviations from the mean occurrence of each error type per question were calculated and visualized using a heatmap. All statistical analyses and visualizations were performed in R version 4.5.1. Data handling and plotting were conducted using tidyverse version 2.0.0, the figures (diverging bar, bubble, percentage histogram, heatmap, dot/lollipop, and violin plots) were generated using ggplot2 version 3.5.1. Inferential comparisons used non-parametric tests appropriate for ordinal data. Differences in relevance across the five model-prompt combinations were tested with the Kruskal–Wallis test, followed by Dunn post-hoc tests with Bonferroni correction. Class-level contrasts between general-purpose and biomedical models are reported descriptively (medians and rank-biserial correlation), as each class comprised only two models. The within-model prompt comparison (GPT-4o with LTM *versus* Active Prompting) used the paired Wilcoxon signed-rank test with a protocol-cluster bootstrap; relevance was additionally modelled with an ordinal cumulative-logit mixed-effects model with a random intercept for protocol (fixed effects for configuration, source, and question). Agreement on reasoning concordance was compared between model types using the chi-square test and sensitivity analyses were conducted treating “partly agree” as acceptable or unacceptable.

To assess potential model-family self-evaluation and stylistic-alignment bias in the GPT-4o-assigned relevance scores, we performed a blinded human validation on a stratified random sample. We stratified by the five model–prompt configurations and drew an equal allocation per configuration (70 assessments each; target *n* = 350, ≈20% of the 1,760 available relevance ratings), with items selected at random across the three protocol sources within each configuration. We drew a stratified random sample with equal allocation per configuration (72 labels each; *n* = 360, ≈20% of the 1,809 available labels), selected at random across the three protocol sources. Two pharmacoepidemiologists (MS and XZ), blinded to model identity and to the GPT-4o label, independently judged whether they agreed with the GPT-4o assigned label for each sampled response; non-evaluable items were excluded. Percent agreement (expert-*versus*-expert and human-*versus*-GPT-4o) was reported overall and separately for each evaluated model. Human logic-of-justification ratings were assigned only where the model produced an assessable justification; where the output was absent, inadequate, or unassessable, no human logic score was recorded. Because this missingness is plausibly informative, complete-case human-logic summaries may be biased, and we reported adverse-case sensitivity analyses in which missing or unassessable ratings were treated as failure, together with a best-case bound. Two-sided p-values < 0.05 were considered statistically significant.

### Reporting Standards

This article was prepared in accordance with the TRIPOD-LLM and CHART reporting standards (Supplementary Tables 1 and 2). [24, 25]. All statistical analysis code, study protocols, LLM prompt strategies, relevance and logic of justification assessments are publicly available via a GitHub repository (Link: https://github.com/madelsu/LLM-for-pharmacoepi-study-design.git).

### Use of Large Language Models

Beyond the LLMs that were the object of study, general-purpose LLM assistance was used for language editing of the manuscript and for drafting portions of the R analysis code; all model-generated text and code were reviewed, verified, and edited by the authors, who take full responsibility for the final content.

## Results

### Descriptive Analysis

A total of 46 pharmacoepidemiological protocols were included: 16 from DARWIN EU®, 15 from the HMA-EMA Catalogue, and 15 from the Sentinel System. The 46 protocols are available at https://github.com/madelsu/LLM-for-pharmacoepi-study-design/tree/main/Data. There was full agreement between the human experts when extracting information from the protocols. DARWIN EU® and HMA-EMA protocols involved researchers from Belgium, Estonia, Finland, France, Germany, Italy, the Netherlands, Norway, Romania, Spain, and the United Kingdom, whereas Sentinel protocols originated from the United States. Study topics were primarily drug utilization (*n* = 18) and disease epidemiology (*n* = 15), followed by safety (*n* = 11) and effectiveness (*n* = 2). Across the included protocols, 15 data sources were represented, of which IQVIA Germany (*n* = 23), SIDIAP (*n* = 15), and the Sentinel Distributed Database (*n* = 14) were the most frequently used. Fifteen ontology-code systems were identified, with ICD, SNOMED, ATC, CPT, and RxNorm among the most frequently used for concept definition. Less frequently used systems included MedDRA and Read codes.

Outcome coverage was incomplete and differed by outcome. Relevance ratings were present for 1,058 of 1,242 expected general-purpose assessments (85.2%) and 702 of 828 biomedical assessments (84.8%); GPT-4o-assessed reasoning-concordance labels for 1,107/1,242 (89.1%) and 702/828 (84.8%); and human logic-of-justification ratings for only 838/1,242 (67.5%) and 122/828 (14.7%). The severe, differential missingness was therefore confined to the human logic-of-justification outcome (Supplementary Table 3); the GPT-4o-assigned outcomes had high and comparable coverage across model classes. The protocols were published between 2018 and 2024, with annual frequencies of 1 from 2018, 2 from 2019, 4 from 2020, 3 from 2021, 3 from 2022, 29 from 2023, and 4 from 2024 (Supplementary Table 4).

### Relevance

**Fig. 1:**
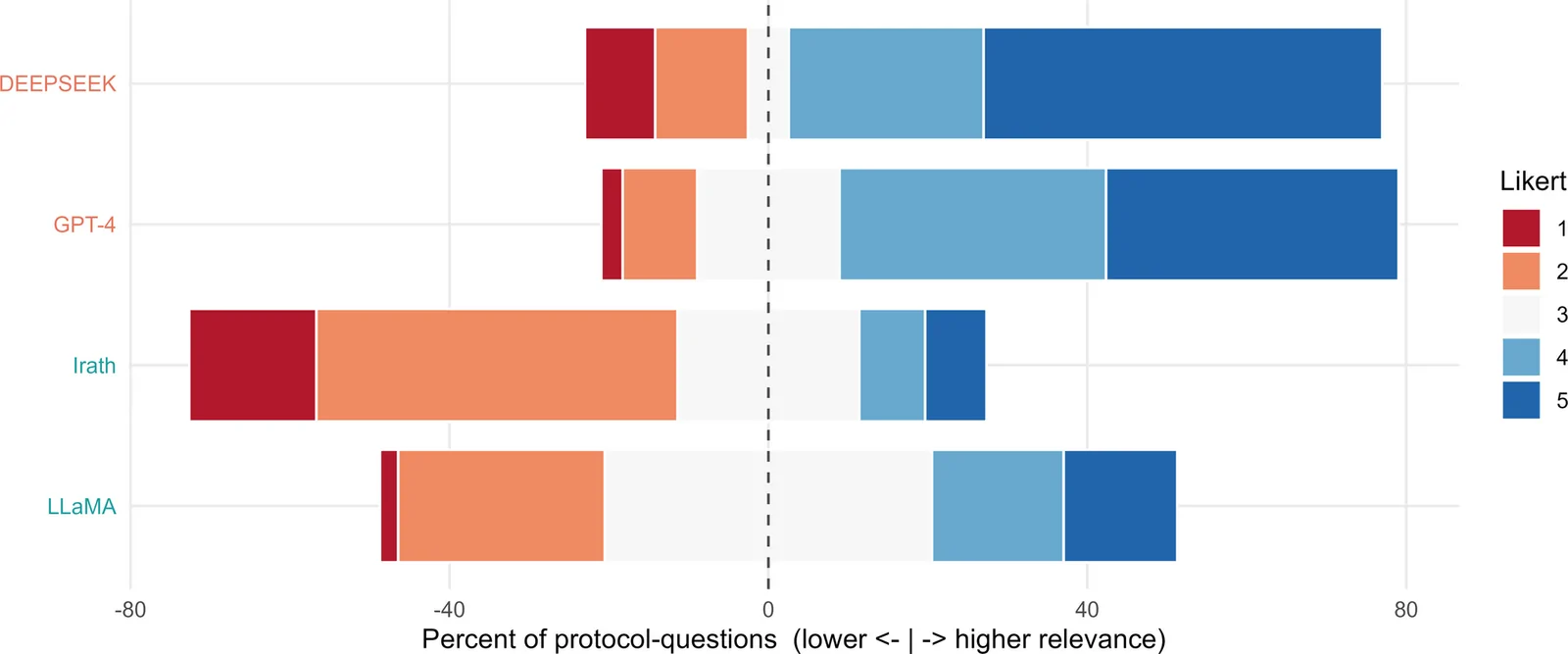
Diverging stacked bar chart of the distribution of 5-point relevance scores for outputs generated by different LLM architectures. Legend: Each horizontal bar represents one LLM architecture and shows the percentage distribution of relevance scores, diverging around the neutral category (score 3); segments to the left correspond to lower scores (1–2) and segments to the right to higher scores (4–5). Colour encodes the relevance level from 1 (completely inaccurate) to 5 (highly accurate).

**Fig. 2:**
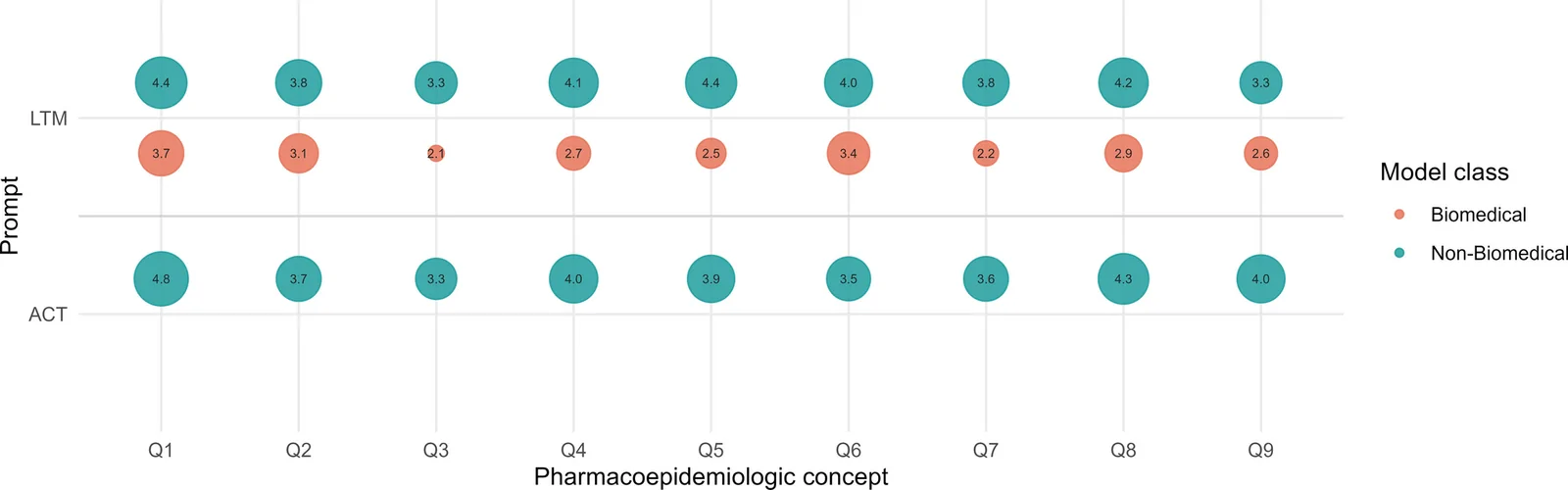
Bubble plot of mean 5-point relevance scores by prompt strategy across the nine pharmacoepidemiologic concepts, faceted by model class. Legend: Each bubble shows the mean relevance score for a given prompt strategy (LTM or ACT) and pharmacoepidemiologic concept (Q1–Q9); bubble size is proportional to the mean and the printed value gives the mean. Panels separate non-biomedical (teal) from biomedical (salmon) LLMs. LTM = Least-to-Most; ACT = Active prompt.

**Fig. 3:**
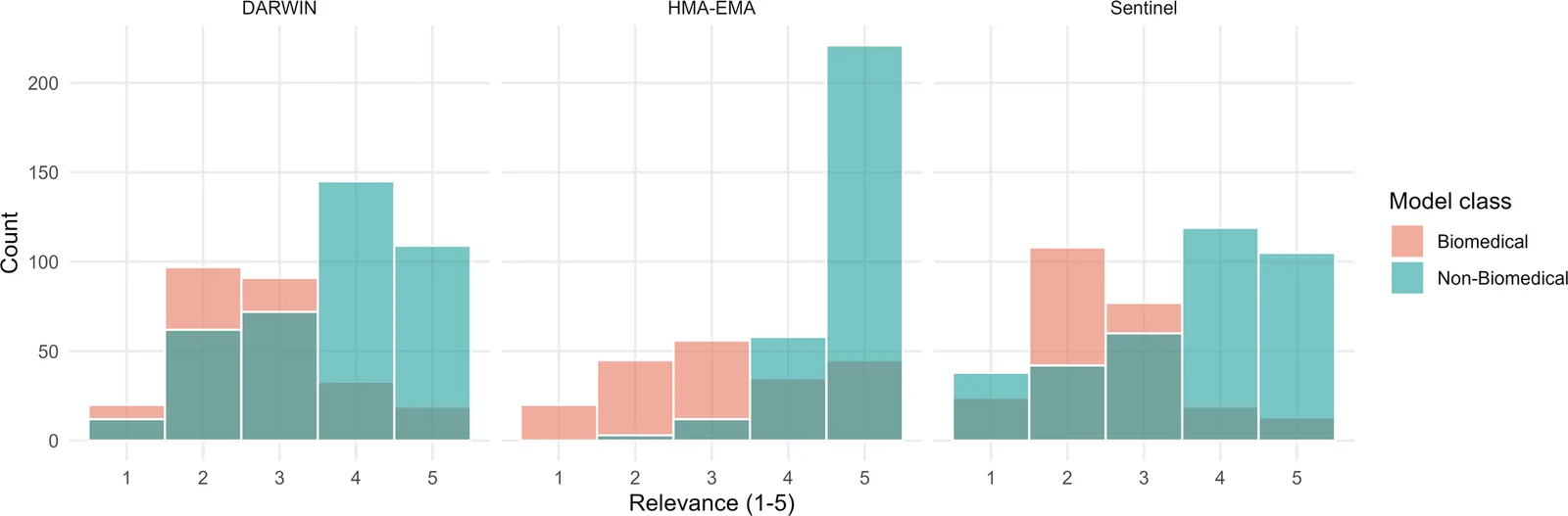
Percentage histogram of 5-point relevance scores across the three pharmacoepidemiologic protocol sources (DARWIN, HMA-EMA, and Sentinel), by model class. Legend: Bars show the percentage of relevance scores at each level (1 = completely inaccurate to 5 = highly accurate) within each protocol source, expressed within each source × model-class group so the two classes are directly comparable. Teal bars denote non-biomedical LLMs and salmon bars denote biomedical LLMs.

Overall, GPT-4o-LTM and DeepSeek-R1-LTM achieved the highest relevance scores. GPT-4o-LTM showed the most stable performance, reaching a median score of 4 with a narrow IQR in 8 of 9 questions for HMA-EMA protocols. Across evaluated concepts, general-purpose LLMs outperformed the biomedical LLMs, with higher median relevance scores and greater overall stability (Fig. 1). Descriptively, differences between LTM and Active Prompting were modest and concept-specific rather than a consistent overall advantage (Fig. 2); in the model-controlled comparison overall relevance did not differ significantly (paired *p* = 0.93). Protocol sources influenced model performance. HMA-EMA protocols yielded the highest and most stable relevance scores, particularly for GPT-4o-LTM and DeepSeek-R1-LTM. Sentinel protocols also supported relatively strong performance but with greater variability. In contrast, DARWIN EU® protocols were associated with lower and less stable relevance, especially for outcome and follow-up definitions. Across all protocol sources, biomedical LLMs showed lower relevance overall, with DARWIN EU® protocols yielding consistently low-scoring outputs (Fig. 3). These differences were statistically significant: relevance differed across the five model-prompt combinations (Kruskal–Wallis H = 384.3, *p* < 0.001), and Dunn post-hoc tests showed that the three general-purpose combinations did not differ from one another (all adjusted *p* = 1.00) but each significantly exceeded both biomedical combinations (all adjusted *p* < 0.001). In an ordinal cumulative-logit mixed-effects model with a random intercept for protocol, both biomedical configurations scored substantially lower than GPT-4o-LTM (BioLlama-LTM *β* = − 1.44 and Qwen2-med-LTM *β* = − 2.73, both *p* < 0.001), Active Prompting did not differ from LTM (*β* = − 0.03, *p* = 0.83), and one general-purpose contrast (DeepSeek-R1-LTM, *β* = 0.30, *p* = 0.044) did not retain statistical significance after adjustment for multiple comparisons. Pooled by model type, general-purpose models scored higher than biomedical models (median 4 vs 3; rank-biserial *r* = 0.51); because each class comprises only two models, this class-level contrast is reported descriptively rather than as an inferential test. When the model was held constant, however, GPT-4o with LTM and GPT-4o with Active Prompting did not differ significantly in overall relevance (paired Wilcoxon *p* = 0.93; unpaired *p* = 0.55), indicating that prompt strategy did not materially affect overall relevance in this model-controlled comparison and contributed less to performance than model type. Of the 350 sampled relevance assessments, 347 were scored by both experts (3 were excluded as non-evaluable). The two blinded pharmacoepidemiologists (MS and XZ) were in complete agreement with each other and with the GPT-4o-assigned relevance scores (percent agreement 100%), and this concordance held for every evaluated model, including the two GPT-4o configurations (100% for each; per-configuration *n* = 70, 70, 70, 67, and 70). The automated GPT-4o relevance ratings were therefore fully reproduced by independent blinded human experts, with no evidence that scores were inflated for GPT-4o-generated responses relative to the other models.

### Logic of Justification

**Fig. 4:**
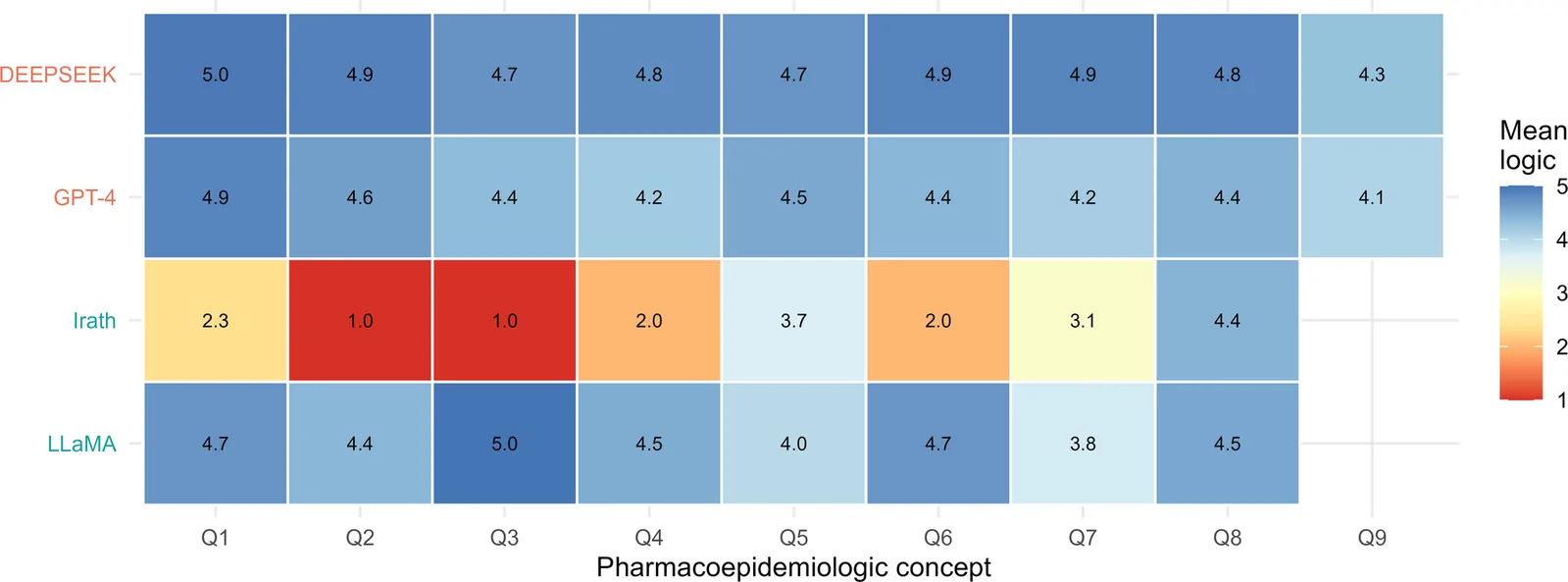
Heatmap of mean 5-point logic-of-justification scores for outputs generated by different LLM architectures across the nine concepts. Legend: Each cell shows the mean logic-of-justification score (1 = completely inaccurate to 5 = highly accurate) for one LLM (rows) and one pharmacoepidemiologic concept (Q1–Q9, columns). Darker teal indicates higher mean scores; a dash (–) marks combinations for which human ratings were not available. The biomedical summaries in Figs. 4, 5 and 6 derive from a small subset (human-logic coverage 14.7%) with substantial and plausibly informative missingness and should be interpreted cautiously.

**Fig. 5:**
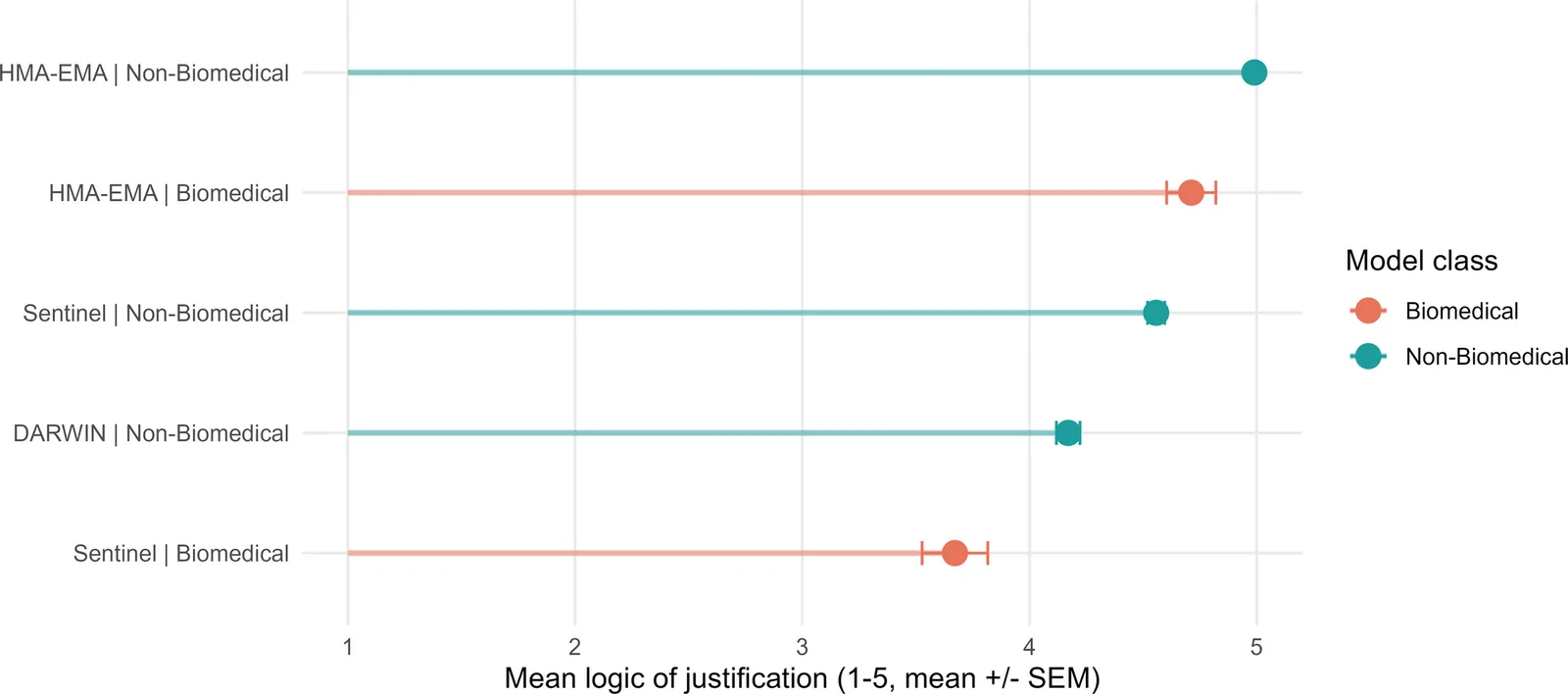
Cleveland dot (lollipop) plot of mean 5-point logic-of-justification scores by protocol source and model class, with standard-error bars. Legend: Each point shows the mean logic-of-justification score (1–5) for a protocol source and model class, with horizontal bars indicating ± one standard error of the mean. Teal points denote non-biomedical LLMs and salmon points denote biomedical LLMs; the DARWIN × biomedical combination is omitted because human ratings were not available. The biomedical summaries in Figs. 4, 5 and 6 derive from a small subset (human-logic coverage 14.7%) with substantial and plausibly informative missingness and should be interpreted cautiously. Because the DARWIN-by-biomedical cells contain no human logic ratings, Fig. 5 is not interpreted as supporting a source-by-model-class difference for those cells.

**Fig. 6:**
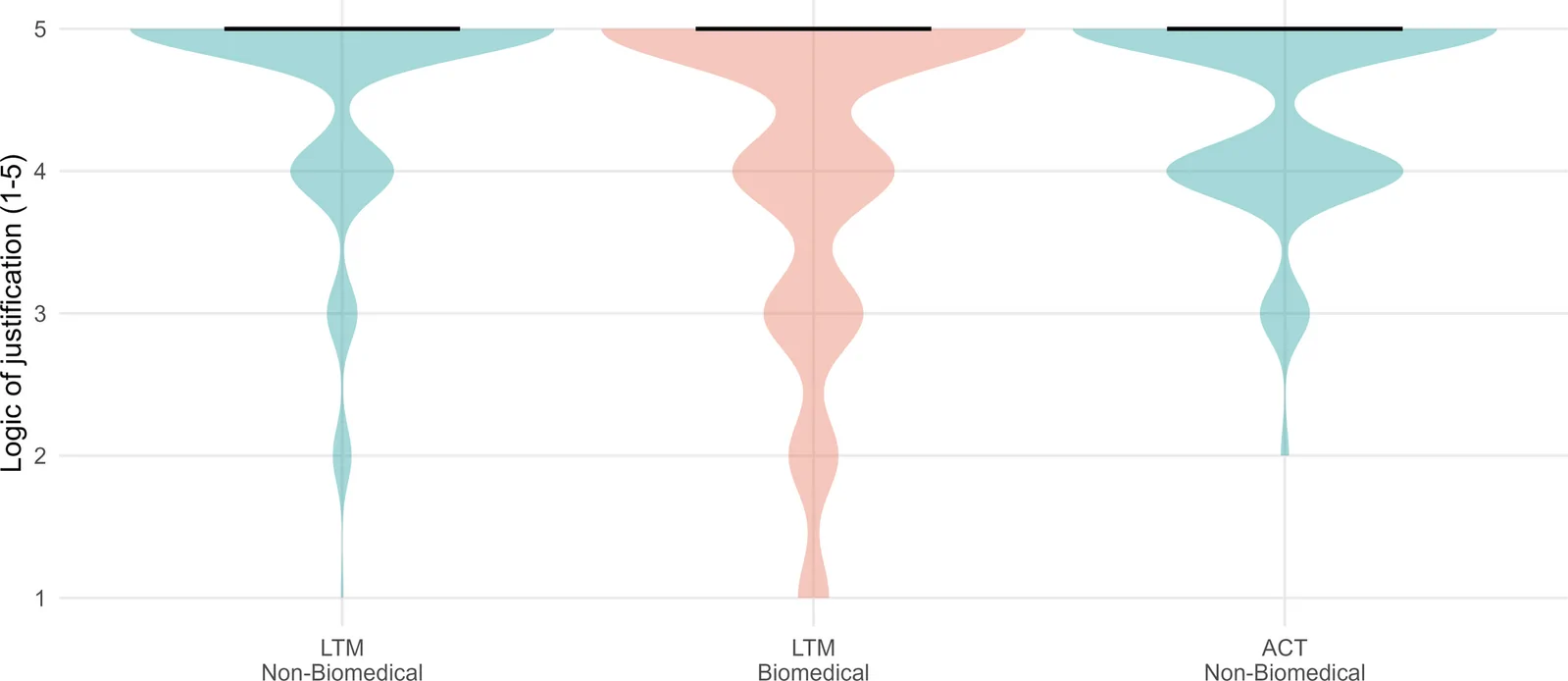
Violin plot with jittered observations of 5-point logic-of-justification scores by prompt strategy and model class. Legend: Violins show the distribution of logic-of-justification scores (1–5) by prompt strategy (LTM or ACT) and model class, with individual ratings overlaid as jittered points and the horizontal line marking the group mean. Teal denotes non-biomedical LLMs and salmon denotes biomedical LLMs. The biomedical summaries in Figs. 4, 5 and 6 derive from a small subset (human-logic coverage 14.7%) with substantial and plausibly informative missingness and should be interpreted cautiously.

The overall pattern for logic of justification largely mirrored the relevance results. GPT-4o-LTM and DeepSeek-R1-LTM again achieved the strongest performance, with GPT-4o-LTM on HMA-EMA protocols reaching the highest and most stable scores across questions. Non-biomedical LLMs generally produced more coherent and methodologically plausible justifications than biomedical LLMs, which were more frequently not assessable because of missing or inadequate reasoning (Fig. 4). As in the relevance analysis, HMA-EMA protocols yielded the most stable performance, whereas Sentinel and DARWIN EU® protocols showed greater variability across concepts (Fig. 5). Any apparent differences between LTM and Active Prompting were not statistically supported in the model-controlled comparison (paired *p* = 0.93) and are reported descriptively. However, LTM also produced occasional low-end outliers, suggesting that errors introduced in early steps could propagate across later dependent responses (Fig. 6). Quantitatively, general-purpose models were rated “agree” on GPT-4o-assessed reasoning concordance, a GPT-4o agreement judgment distinct from the human logic-of-justification ratings, in 70.7% of assessments *versus* 20.2% for biomedical models when “partly agree” responses were excluded from the denominator (chi-square = 406.1, *p* < 0.001; the difference remained large under sensitivity analyses treating “partly agree” as acceptable, 73.6% vs 24.1%, or as unacceptable, 63.7% vs 19.2%). The general-purpose advantage in reasoning concordance was likewise preserved under protocol-level clustering (biomedical class *β* = − 2.52, *p* < 0.001). In the reasoning-concordance validation (*n* = 360; 72 per configuration), the two experts were in complete agreement with each other and with the GPT-4o-assigned labels (percent agreement 100%), consistently across all five evaluated models. The GPT-4o reasoning-concordance labels were thus reproduced by independent blinded human classification, indicating that the marked general-purpose-*versus*-biomedical difference in reasoning concordance (70.7% vs 20.2%) is not an artefact of GPT-4o self-evaluation. Under complete-case analysis the human logic scores were similar across classes (general-purpose 4.56 *versus* biomedical 4.16); however, treating missing or unassessable ratings as failure widened the difference substantially (3.40 *versus* 1.47), while a best-case bound gave 4.70 *versus* 4.88. Because the biomedical models had far greater missingness, the complete-case comparison is conservative with respect to the general-purpose advantage in logic of justification.

### Ontology-Code Agreement

We classified every non-matching model output for the ontology-code task (364 errors across the three model–prompt configurations) into the six categories (Supplementary Table 5). Of 477 model-generated code mappings, 113 matched the reference and 364 were errors (Supplementary Table 6). Errors were dominated by omission for 268/364 (73.6%), which means that the model missed codes identified by experts, and by mapping to a coding system other than the one used operationally by the protocol (62/364, 17.0%). The latter arose almost entirely for SNOMED and RxNorm concepts, where the models returned well-formed native SNOMED CT and RxNorm identifiers while the reference concept sets are expressed as OMOP standard concept IDs. The remaining categories were infrequent: overly broad or parent-level substitutions (9/364, 2.5%), well-formed codes in the correct terminology that nonetheless did not relate to the one mentioned in the reference set (18/364, 4.9%), malformed or non-existent codes (5/364, 1.4%), and single instances of ingredient-*versus*-product and clinical-*versus*-administrative confusion. Set-based metrics (precision, recall, F1, Jaccard and exact-set agreement) are reported by configuration and coding system (e.g., GPT-4o-LTM precision 0.52, recall 0.28, F1 0.33, exact-set 12.5%; ICD F1 0.50, ATC/CPT/HCPCS F1 0.32–0.37). Native SNOMED CT and RxNorm identifiers evaluated against OMOP standard concept IDs were reported separately as coding-convention differences rather than errors.

### Error Classification

Error analysis showed that the biomedical LLMs were particularly prone to recurrent structured failures. The most frequent error types were irrelevant auto-generated questions (*n* = 189) and lack of justification (*n* = 105), followed by long answers (*n* = 36) and non-adherence to the intended algorithm (*n* = 11). Less frequent errors included prompt echoing, model crash, and language inconsistency. Overall, QuantFactory/Bio-Medical-Llama-3-8B showed more concentrated error clusters across core questions, particularly Questions 3–7, whereas Qwen displayed a flatter but still suboptimal error profile (Supplementary Fig. 1). See Supplementary Table 7 for error definitions and examples.

## Discussion

The use of LLMs in pharmacoepidemiology has received increasing research attention in recent years [11]. Despite this growing interest, important concerns remain regarding the reliability of general-purpose LLMs, the suitability of their training data for scientific and regulatory tasks, and the performance of newly released models and prompt-engineering strategies in supporting pharmacoepidemiologic study design. These uncertainties highlight the need for systematic evaluation of LLM capabilities in this domain. This study addressed that need by evaluating off-the-shelf general-purpose LLMs, biomedical LLMs, and advanced prompt-engineering strategies for support of pharmacoepidemiologic study design. A total of 46 study protocols from DARWIN EU®, the Sentinel System, and HMA-EMA were assessed by both human experts and LLMs. Performance was assessed across multiple dimensions, including relevance, logic of justification, ontology-code agreement, and error patterns.

The main finding was that general-purpose LLMs, particularly GPT-4o and DeepSeek-R1 paired with LTM prompting, consistently outperformed the biomedical LLMs evaluated in this study. This pattern was observed not only for the primary outcome of relevance, but also for logic of justification, suggesting that the advantage of these LLMs extended beyond answer selection to broader methodological reasoning. Although the biomedical LLMs were domain-specific, they showed lower relevance, weaker justification quality, and more frequent structured failures, particularly irrelevant auto-generated questions and lack of justification. This suggests that domain adaptation alone may be insufficient for tasks that require not only biomedical knowledge but also structured interpretation of study design elements such as index date, follow-up, covariates, and outcome definitions. In other words, the task appears to depend as much on methodological reasoning and contextual interpretation as on domain vocabulary. This is consistent with findings reported by Heckmann *et al*. in pharmacovigilance, where general-purpose LLMs also outperformed biomedical LLMs on tasks requiring structured reasoning and explainable output. [26] It is also in line with Dada *et al*., who showed that increasing biomedical specialization does not necessarily translate into better performance on medical tasks and may come at the cost of instruction-following or broader task adaptability. [27] Together, these findings suggest that pharmacoepidemiologic study design is not simply a knowledge retrieval task, but one that requires LLMs to understand instructions and organize interrelated pharmacoepidemiological design choices in a coherent way. The stronger performance of general-purpose LLMs therefore suggests that, for this type of task, broader semantic understanding may currently be more important than narrow biomedical tuning. These model groups, however, differed not only in domain specialization but also in parameter scale, architecture, instruction-tuning, and training maturity; the contrast therefore reflects a comparison between frontier-scale general-purpose systems and smaller open biomedical models rather than a clean test of biomedical specialization, and the conclusion is framed accordingly.

Several non-mutually exclusive mechanisms may explain the stronger performance of the general-purpose models. First, scale and pretraining breadth: GPT-4o and DeepSeek-R1 are frontier-scale systems trained on far larger and more diverse corpora than the 8-billion-parameter Bio-Medical-Llama and the 1.5-billion-parameter Qwen medical fine-tune, which plausibly supports stronger general reasoning. Second, instruction-tuning and alignment maturity: the dominant biomedical failure modes were irrelevant auto-generated questions and lack of justification, that is, instruction-following failures rather than knowledge gaps, and these are precisely the behaviours that large-scale instruction-tuning and reinforcement learning from human feedback are designed to suppress. Third, a specialization trade-off, whereby narrow biomedical fine-tuning of small base models can erode general instruction-following and reasoning, consistent with Dada *et al*. [27] Disentangling these mechanisms from domain specialization itself would require comparisons across matched model scales, which the present design did not permit.

Any differences between LTM and Active Prompting were concept-specific and modest rather than a consistent overall advantage; in the one model-controlled comparison (GPT-4o with LTM *versus* Active Prompting), overall relevance did not differ significantly (paired *p* = 0.93), and prompt comparisons are therefore reported descriptively. This likely reflects the advantage of decomposing complex pharmacoepidemiologic reasoning into sequential subtasks, allowing LLMs to build more complete and coherent reasoning chains. For tasks that involve multiple dependent design elements, such structured prompting may be particularly useful. At the same time, the persistence of low-end outliers indicates that this structure may also increase sensitivity to early-step errors, which can then propagate into later responses. Prompting therefore improved performance but did not remove the need for caution or review. This is especially relevant in workflows where later answers depend directly on earlier ones, as was the case for several of the pharmacoepidemiologic concepts assessed in this study. This advantage was, however, concept-specific: in the one comparison that held the model constant (GPT-4o with LTM *versus* Active Prompting), overall relevance did not differ significantly. Because the two strategies also differ in length, density, and specificity, residual prompt-content confounding cannot be excluded, and a fully length- and content-matched comparison of prompting strategies remains an important direction for future work.

A notable finding was the influence of protocol source. HMA-EMA protocols yielded the highest and most stable scores, while Sentinel protocols showed broader variability, and DARWIN EU® protocols tended to produce lower and less stable performance. Because the models did not receive the protocol document, these source differences cannot be attributed to protocol formatting and more plausibly reflect differences in topic mix, title informativeness, and reference specificity across the three catalogues. This suggests that LLM outputs are influenced not only by model architecture and prompting strategy, but also by the clarity, consistency, and reporting style of the source material. Consistently structured and informative protocol metadata may nonetheless reduce ambiguity for both human and machine readers. More consistently structured protocols may reduce ambiguity for both human and machine readers and may be especially important in highly regulated settings, including EMA-related contexts, where documentation quality, traceability, and methodological consistency are central requirements.

Ontology coding remained a clear weakness across all LLMs and protocol sources. Even when the LLMs performed well on narrative design tasks, ontology-code agreement was generally limited, with near-zero agreement for SNOMED CT and RxNorm largely reflecting a coding-convention difference (native identifiers *versus* OMOP standard concept IDs) rather than outright errors, whereas ICD showed comparatively higher agreement. This suggests that coding tasks place different demands on LLMs than narrative interpretation tasks and that good performance in one area does not necessarily translate into reliable performance in the other. This distinction is important because ontology coding is not a minor technical detail in pharmacoepidemiologic work. It is part of how exposure, outcome, and covariate definitions become operationalizable and reproducible. Poor coding performance therefore has direct implications for downstream validity. This means that although LLMs may assist with interpretation and drafting, ontology coding should not rely on free-text inference alone. Deterministic support, such as curated dictionaries, crosswalks, or rule-based validation, remains necessary.

### Strengths and Limitations

A key strength of this study is its systematic comparison across multiple dimensions, including model architecture, prompt strategy, protocol source, and ontology coding. This multidimensional framework provides a more comprehensive picture of LLM behavior in pharmacoepidemiologic contexts than would be possible from isolated performance metrics alone. Another strength is the use of human expert ratings as a benchmark, which grounds the findings in practical relevance and helps identify where automation may meaningfully support expert workflows and where risks remain. The inclusion of protocols from three major networks, DARWIN EU®, HMA-EMA, and Sentinel, further strengthens the study by allowing performance to be examined across diverse reporting standards and coding systems. In addition, the focus on both relevance and logic of justification extends the evaluation beyond surface-level accuracy and addresses the interpretability and soundness of model reasoning.

Several limitations should nevertheless be acknowledged. First, the study evaluated off-the-shelf LLMs without task-specific fine-tuning. While this reflects real-world use cases, performance may differ with domain-adapted training pipelines incorporating structured ontology coding. Second, the evaluation of logic of justification showed substantial variability, particularly for complex concepts, suggesting that the scoring framework may require further refinement to capture nuance reliably. Third, although protocols from three networks were included, they are not fully representative of all global pharmacoepidemiologic practices, and results may differ in settings with alternative reporting standards or coding systems. Finally, the reliance on Likert-scale ratings and agreement thresholds introduces subjectivity into the evaluation, even though multiple raters and comparisons were used to mitigate this limitation. Fifth, the general-purpose and biomedical models differed substantially in scale, architecture, and instruction-tuning, so the comparison should be read as contrasting readily deployable options rather than isolating biomedical specialization; matched-scale general-purpose models, larger biomedical models, and retrieval-augmented biomedical systems are needed to disentangle these factors. Sixth, the screening that reduced the initial 42 combinations to five was based on only three protocols, so a marginally competitive combination could in principle have been excluded. Seventh, the Least-to-Most and Active prompts differed in length and informational content, leaving open the possibility that prompt content rather than prompting strategy contributed to some observed differences.

Additional limitations concern the evaluation itself. Relevance and reasoning-concordance outcomes were assigned by GPT-4o, including for GPT-4o's own outputs. We addressed the resulting risk of model-family self-evaluation and stylistic-alignment bias through a blinded human validation on a stratified sample, in which two pharmacoepidemiologists reproduced the GPT-4o scores with 100% agreement for every evaluated model; residual limitations are that the original human logic ratings were a two-rater consensus rather than independent double-rating, and that the validation sample, though stratified, was limited in size. The human logic-of-justification outcome had substantial and differential missingness (67.5% coverage for general-purpose *versus* 14.7% for biomedical configurations), so Figs. 4, 5 and 6 should be interpreted with caution and no source-by-model-class inference is drawn where a cell contains no human data. The primary outcome measures concordance with historical reference protocols rather than general study-design validity, and a divergent answer may be methodologically acceptable yet score poorly against the historical reference. For the ontology task, native SNOMED CT and RxNorm identifiers evaluated against OMOP-concept-ID references reflect coding-convention differences that require a prespecified terminology crosswalk to score, and the design cannot separate mapping errors from upstream concept identification. No pre-specified concept-frequency measure was available in the reference set, so common-*versus*-rare concept stratification was not performed. All models were used off-the-shelf without task-specific fine-tuning: the general-purpose models (GPT-4o, snapshot gpt-4o-2024–08-06 with an October 2023 knowledge cutoff, and DeepSeek-R1) were accessed through hosted APIs at full precision, whereas the retained Bio-Medical-Llama-3-8B was run as a GGUF-quantized build (QuantFactory/Bio-Medical-Llama-3-8B-GGUF, Q4_K_M) and Qwen2-1.5B-medical from its public checkpoint; this precision and quantization asymmetry is a further reason the comparison reflects readily deployable configurations rather than model class per se. Because inference stack, numerical precision, quantization, model size, and instruction-tuning maturity differed across models, the comparison reflects readily deployable configurations rather than a controlled contrast of biomedical specialization; no causal effect of biomedical tuning can be inferred. Memorization therefore cannot be excluded as a contributor to concordance, and we identify a prospective evaluation using post-cutoff protocols as a stronger future test. Under Active prompting, the Q4 and Q10 prompt fields referencing concepts from earlier questions were left empty (the model's own prior answers were not supplied), so Active prompting was independent per question and, unlike Least-to-Most, not subject to error propagation. A stronger future benchmark would compare general-purpose and biomedical-tuned models matched on base architecture, size, inference stack, quantization, context budget, and prompting protocol, with retrieval-augmented variants evaluated separately.

## Conclusions

This study showed that off-the-shelf general-purpose LLMs, particularly GPT-4o and DeepSeek-R1 combined with LTM prompting, outperformed the biomedical LLMs evaluated in this study for support of pharmacoepidemiologic study design. Their advantage was evident not only for the primary outcome of relevance, but also for logic of justification, indicating that stronger performance was observed both in alignment with protocol-derived expert answers and in the ability to provide more coherent methodological reasoning. Given the variability across protocol sources, the dependence on prompt strategy, the subjectivity of the Likert ratings, and the poor ontology-code performance, these systems are not yet reliable for autonomous pharmacoepidemiologic study design. They are best positioned as expert-assistive tools that can support the drafting and summarizing of individual design elements under the review of a qualified pharmacoepidemiologist, rather than as automated design systems. Because the biomedical models evaluated were also smaller and less instruction-tuned than the general-purpose models, the comparison reflects currently deployable options rather than biomedical specialization per se.

## Supplementary Information

Below is the link to the electronic supplementary material.

ESM 1 (DOCX 280 KB)

ESM 2 (DOCX 21.0 KB)

## Data Availability

All statistical analysis code, study protocols, LLM prompt strategies, relevance and logic of justification assessments, and LLM-generated outputs used in this study are publicly available via GitHub (https://github.com/madelsu/LLM-for-pharmacoepi-study-design.git). The pharmacoepidemiological protocols analysed during the current study are available in the same repository at https://github.com/madelsu/LLM-for-pharmacoepi-study-design/tree/main/Data.
