# Supplementary material for: Employing General-Purpose and Biomedical Large Language Models with Advanced Prompt Engineering for Pharmacoepidemiologic Study Design
Source: Pharm Res. 2026 Sep 11;43(10):3701–15. doi: 10.1007/s11095-026-04190-5 (PMC13736468; doi:10.1007/s11095-026-04190-5)
Supplement: Supplementary file 1 [file 11095_2026_4190_MOESM1_ESM.docx]

**SUPPLEMENTARY MATERIAL**

**Employing General-Purpose and Biomedical Large Language Models with Advanced Prompt Engineering for Pharmacoepidemiologic Study Design**

**Metrics**

Manuscript: 5725

Abstract: 223

References: 27

Figures and tables: 7 tables and 1 figure

**Supplementary Table 1.** TRIPOD-LLM Checklist.

| **Section** | **Item** | **Description** | **Page no.** |
| --- | --- | --- | --- |
| **Title** | 1 | Identify the study as developing, fine-tuning and/or evaluating the performance of an LLM, specifying the task, the target population and the outcome to be predicted. | 1 |
| **Introduction** |  |  |  |
| **Abstract** | 2 | See TRIPOD-LLM for abstracts. | 3 |
| **Background** | 3a | Explain the healthcare context/use case (for example, administrative, diagnostic, therapeutic and clinical workflow) and rationale for developing or evaluating the LLM, including references to existing approaches and models. | 4 |
|  | 3b | Describe the target population and the intended use of the LLM in the context of the care pathway, including its intended users in current gold standard practices (for example, healthcare professionals, patients, public or administrators). | 4 |
| **Objectives** | 4 | Specify the study objectives, including whether the study describes the initial development, fine-tuning or validation of an LLM (or multiple stages). | 4 |
| **Methods** |  |  |  |
| **Data** | 5a | Describe the sources of data separately for the training, tuning and/or evaluation datasets and the rationale for using these data (for example, web corpora, clinical research/trial data, EHR data or unknown). | 5 |
|  | 5b | Describe the relevant data points and provide a quantitative and qualitative description of their distribution and other relevant descriptors of the dataset (for example, source, languages and countries of origin). | 5,8 |
|  | 5c | Specifically state the date of the oldest and newest item of text used in the development process (training, fine-tuning and reward modeling) and the evaluation datasets. | 5 |
|  | 5d | Describe any data preprocessing and quality checking, including whether this was similar across text corpora, institutions and relevant sociodemographic groups. | 5-6 |
|  | 5e | Describe how missing and imbalanced data were handled and provide reasons for omitting any data. | NA |
| **Analytical Methods** | 6a | Report the LLM name, version and last date of training. | 6 |
|  | 6b | Report details of the LLM development process, such as LLM architecture, training, fine-tuning procedures and alignment strategy (for example, reinforcement learning and direct preference optimization) and alignment goals (for example, helpfulness, honesty and harmlessness). | 6 |
|  | 6c | Report details of how the text was generated using the LLM, including any prompt engineering (including consistency of outputs), and inference settings (for example, seed, temperature, max token length and penalties), as relevant. | 6 |
|  | 6d | Specify the initial and postprocessed output of the LLM (for example, probabilities, classification and unstructured text). | 7 |
|  | 6e | Provide details and rationale for any classification and, if applicable, how the probabilities were determined and thresholds identified. | 7 |
| **LLM Output** | 7a | Include metrics that capture the quality of generative outputs, such as consistency, relevance, accuracy and presence/type of errors compared to gold standards. | 9-10, 23-29 |
|  | 7b | Report the outcome metrics’ relevance to the downstream task at deployment time and, where applicable, the correlation of metric to human evaluation of the text for the intended use. | 6,8, 23-29 |
|  | 7c | Clearly define the outcome, how the LLM predictions were calculated (for example, formula, code, object and API), the date of inference for closed-source LLMs and evaluation metrics. | 6-7 |
|  | 7d | If outcome assessment requires subjective interpretation, describe the qualifications of the assessors, any instructions provided, relevant information on demographics of the assessors and inter-assessor agreement. | 6 |
|  | 7e | Specify how performance was compared to other LLMs, humans and other benchmarks or standards. | 6,8-10 |
| **Annotation** | 8a | If annotation was done, report how the text was labeled, including providing specific annotation guidelines with examples. | 6 |
|  | 8b | If annotation was done, report how many annotators labeled the dataset(s), including the proportion of data in each dataset that was annotated by more than one annotator, and the inter-annotator agreement. | 6 |
|  | 8c | If annotation was done, provide information on the background and experience of the annotators or the characteristics of any models involved in labeling. | 6 |
| **Prompting** | 9a | If research involved prompting LLMs, provide details on the processes used during prompt design, curation and selection. | 6 |
|  | 9b | If research involved prompting LLMs, report what data were used to develop the prompts. | 6 |
| **Summariza-tion** | 10 | Describe any preprocessing of the data before summarization. | NA |
| **Instruction tuning/ alignment** | 11 | If instruction tuning/alignment strategies were used, what were the instructions, data and interface used for evaluation, and what were the characteristics of the populations doing the evaluation? | NA |
| **Compute** | 12 | Report compute, or proxies thereof (for example, time on what and how many machines, cost on what and how many machines, inference time, floating-point operations per second), required to carry out methods. | NA |
| **Ethical approval** | 13 | Name the institutional research board or ethics committee that approved the study and describe the participant-informed consent or the ethics committee waiver of informed consent. | 15 |
| **Open science** | 14a | Give the source of funding and the role of the funders for the present study. | 15 |
|  | 14b | Declare any conflicts of interest and financial disclosures for all authors. | 15 |
|  | 14c | Indicate where the study protocol can be accessed or state that a protocol was not prepared. | 15 |
|  | 14d | Provide registration information for the study, including register name and registration number, or state that the study was not registered. | 15 |
|  | 14e | Provide details of the availability of the study data. | 15 |
|  | 14f | Provide details of the availability of the code to reproduce the study results. | 15 |
| **Public involvement** | 15 | Provide details of any patient and public involvement during the design, conduct, reporting, interpretation or dissemination of the study or state no involvement. | NA |
| **Results** |  |  |  |
| **Participants** | 16a | When using patient/EHR data, describe the flow of text/EHR/patient data through the study, including the number of documents/questions/participants with and without the outcome/label and follow-up time as applicable. | NA |
|  | 16b | When using patient/EHR data, report the characteristics overall and for each data source or setting and development/evaluation splits, including the key dates, key characteristics and sample size. | NA |
|  | 16c | For LLM evaluation that includes clinical outcomes, show a comparison of the distribution of important clinical variables that may be associated with the outcome between development and evaluation data, if available. | NA |
|  | 16d | When using patient/EHR data, specify the number of participants and outcome events in each analysis (for example, for LLM development, hyperparameter tuning and LLM evaluation). | NA |
| **Performance** | 17 | Report LLM performance according to prespecified metrics (see item 7a) and/or human evaluation (see item 7d). | 8-10 |
| **LLM updating** | 18 | If applicable, report the results from any LLM updating, including the updated LLM and subsequent performance. |  |
| **Discussion** | | | |
| **Interpretation** | 19a | Give an overall interpretation of the main results, including issues of fairness in the context of the objectives and previous studies. | 11-13 |
| **Limitations** | 19b | Discuss any limitations of the study and their effects on any biases, statistical uncertainty and generalizability. | 14 |
| **Usability of the LLM in context** | 19c | Describe any known challenges in using data for the specified task and domain context with reference to representation, missingness, harmonization and bias. | 14 |
|  | 19d | Define the intended use for the implementation under evaluation, including the intended input, end-user and level of autonomy/human oversight. | 4 |
|  | 19e | If applicable, describe how poor quality or unavailable input data should be assessed and handled when implementing the LLM; that is, what is the usability of the LLM in the context of current clinical care. | 14 |
|  | 19f | If applicable, specify whether users will be required to interact in the handling of the input data or use of the LLM, and what level of expertise is required of users. | NA |
|  | 19g | Discuss any next steps for future research, with a specific view of the applicability and generalizability of the LLM. | 14 |

*Legend: M = LLM methods; D = de novo LLM development; E = LLM evaluation; H = LLM evaluation in healthcare settings; C = classification; OF = outcome forecasting; QA = long-form question answering; IR = information retrieval; DG = document generation; SS = summarization and simplification; MT = machine translation; API = application programming interface.*

**Supplementary Table 2.** CHART Checklist.

| **Heading** | **No** | **Chart checklist item** | **Page no.** |
| --- | --- | --- | --- |
| **Title and Abstract** | | |  |
| **Title** | 1a | State that the study is assessing one or more generative AI-driven chatbots for clinical evidence or health advice. | 1 |
| **Abstract/ Summary** | 1b | Apply a structured format, if applicable. | 2 |
| **Introduction** | | |  |
| **Background** | 2a | State the scientific background, rationale, and healthcare context for evaluating the generative AI-driven chatbot(s), referencing relevant literature when applicable. | 4 |
|  | 2b | State the aims and research questions including the target audience, intervention, comparator(s), and outcome(s). | 4 |
| **Methods** | | |  |
| **Model Identifiers** | 3a | State the name and version identifier(s) of the generative AI model(s) and chatbot(s) under evaluation, as well as their date of release or last update. | 6 |
|  | 3b | State whether the generative AI model(s) and chatbot(s) are open-source or closed-source/proprietary. | 6 |
| **Model Details** | 4a | State whether the generative AI model was a base model or a novel base model, tuned model, or fine-tuned model. | 6 |
|  | 4b | If a base model is used, cite its development in sufficient detail to identify the model. | 6 |
|  | 4c | If a novel base model, tuned model, or fine-tuned model is used, describe the pre- and/or post-implementation/deployment data and parameters. | NA |
| **Prompt Engineering** | 5a | Describe the evolution of study prompt development. | 6 |
|  | 5ai | Describe the sources of prompts. | 6 |
|  | 5aii | State the number and characteristics of the individual(s) involved in prompt engineering. | NA |
|  | 5aiii | Provide details of any patient and public involvement during prompt engineering. | NA |
|  | 5b | Provide study prompts. | 8 |
| **Query Strategy** | 6a | State route of access to generative AI model. | 8 |
|  | 6b | State the date(s) and location(s) of queries for the generative AI-driven chatbot(s) including the day, month, and year as well as city and country. | NA |
|  | 6c | Describe whether prompts were input into separate chat session(s). | NA |
|  | 6d | Provide all generative AI-driven chatbot output/responses | 8 |
| **Prompting** | 7a | Define the ground truth or reference standard used to define successful generative AI-driven chatbot performance. | 6 |
|  | 7b | Describe the process undertaken for generative AI-driven chatbot performance evaluation. | 7 |
|  | 7bi | State the number and characteristics of team members involved in performance evaluation. | 6 |
|  | 7bii | Provide details of any patients and public involvement during the evaluation process. | NA |
|  | 7biii | State whether evaluators were blinded to the identity of the generative AI-driven chatbot(s) under assessment. | NA |
| **Sample Size** | 8 | Report how the sample size was determined. | 5 |
| **Data Analysis** | 9a | Describe statistical analysis methods, including any evaluation of reproducibility of generative AI-driven chatbot responses. | 7 |
|  | 9ai | Report the measures used for performance evaluation. | 8 |
| **Results** | | |  |
|  | 10a | Report the performance evaluation undertaken including the alignment between generative AI-driven chatbot output and ground truth or reference standard using quantitative or mixed methods approaches as applicable. | 8-10, 23-29 |
|  | 10b | For responses deviating from the ground truth or reference standard, state the nature of the difference(s). | 8-10, 23-29 |
|  | 10c | Indicate where the study protocol can be accessed or state that a protocol was not prepared. |  |
| **Discussion** | | |  |
|  | 11a | Interpret study findings in the context of relevant evidence. | 10-14 |
|  | 11b | Describe the strengths and limitations of the study. | 14 |
|  | 11c | Describe the potential implications for practice, education, policy, regulation, and research. | 4 |
| **Open Science** | | |  |
| **Disclosures** | 12a | Report any relevant conflicts of interest for all authors. | 15 |
| **Funding** | 12b | Report sources of funding and their role in the conduct and reporting of the study. | 15 |
| **Ethics** | 12c | Describe the process undertaken for ethical approval. | 15 |
|  | 12ci | Describe the measures taken to safeguard data privacy of patient health information, as applicable. | 15 |
|  | 12cii | State whether permission/licensing was obtained for the use of original, copyrighted data. | 15 |
| **Protocol** | 12d | Provide a study protocol. | 8 |
| **Data availability** | 12e | State where study data, code repository, and model parameters can be accessed. | 8 |

**Supplementary Table 3.** Data flow and outcome coverage for every model–prompt–source combination.

| **Configuration** | **Class** | **Source** | **Expected** | **Relevance present** | **Relevance missing** | **Reasoning present** | **Reasoning missing** | **Human-logic present** | **Human-logic missing** |
| --- | --- | --- | --- | --- | --- | --- | --- | --- | --- |
| GPT-4o-LTM | General-purpose | DARWIN | 144 | 140 | 4 | 143 | 1 | 132 | 12 |
| GPT-4o-LTM | General-purpose | HMA-EMA | 135 | 92 | 43 | 126 | 9 | 87 | 48 |
| GPT-4o-LTM | General-purpose | Sentinel | 135 | 122 | 13 | 134 | 1 | 0 | 135 |
| GPT-4o-ACT | General-purpose | DARWIN | 144 | 130 | 14 | 130 | 14 | 129 | 15 |
| GPT-4o-ACT | General-purpose | HMA-EMA | 135 | 101 | 34 | 101 | 34 | 100 | 35 |
| GPT-4o-ACT | General-purpose | Sentinel | 135 | 121 | 14 | 121 | 14 | 117 | 18 |
| DeepSeek-R1-LTM | General-purpose | DARWIN | 144 | 130 | 14 | 130 | 14 | 56 | 88 |
| DeepSeek-R1-LTM | General-purpose | HMA-EMA | 135 | 101 | 34 | 101 | 34 | 99 | 36 |
| DeepSeek-R1-LTM | General-purpose | Sentinel | 135 | 121 | 14 | 121 | 14 | 118 | 17 |
| Qwen2-med-LTM | Biomedical | DARWIN | 144 | 130 | 14 | 130 | 14 | 0 | 144 |
| Qwen2-med-LTM | Biomedical | HMA-EMA | 135 | 100 | 35 | 100 | 35 | 10 | 125 |
| Qwen2-med-LTM | Biomedical | Sentinel | 135 | 121 | 14 | 121 | 14 | 19 | 116 |
| BioLlama-LTM | Biomedical | DARWIN | 144 | 130 | 14 | 130 | 14 | 0 | 144 |
| BioLlama-LTM | Biomedical | HMA-EMA | 135 | 101 | 34 | 101 | 34 | 49 | 86 |
| BioLlama-LTM | Biomedical | Sentinel | 135 | 120 | 15 | 120 | 15 | 44 | 91 |
| General-purpose (3 configs) |  |  | 1242 | 1058 | 184 | 1107 | 135 | 838 | 404 |
| Biomedical (2 configs) |  |  | 828 | 702 | 126 | 702 | 126 | 122 | 706 |

***Legend****: Expected = number of pre-specified protocols (DARWIN 16, HMA-EMA 15, Sentinel 15) × 9 questions. For each outcome the number of ratings present and missing is shown. Relevance and reasoning-concordance were assigned by GPT-4o as a single automated evaluator; human logic-of-justification was assigned by two pharmacoepidemiologists in consensus. All worksheets are restricted to the pre-specified protocol count, so spurious rows beyond the 46 protocols (e.g., a 16th row in the Sentinel–BioLlama sheet) are excluded from every count. Relevance and reasoning-concordance have high, comparable coverage across model classes; the severe differential missingness is confined to the human logic-of-justification outcome (general-purpose 838/1,242, 67.5%; biomedical 122/828, 14.7%).* *The 'missing' column aggregates outputs that were not generated, technically failed, or were not assessable; because the released workbooks do not code the reason for each missing cell, these categories are not separated, and the adverse-case bound treats all of them as failure.*

**Supplementary Table 4.** Protocol release dates.

| **Source** | **Protocol** | **Release date** | **Date source** |
| --- | --- | --- | --- |
| darwin | DARWIN_1 | 2021-02 | HMA-EMA catalogue export |
| darwin | DARWIN_10 | 2023-07 | HMA-EMA catalogue export |
| darwin | DARWIN_11 | 2023-07 | HMA-EMA catalogue export |
| darwin | DARWIN_12 | 2023-01 | HMA-EMA catalogue export |
| darwin | DARWIN_13 | 2023-02 | HMA-EMA catalogue export |
| darwin | DARWIN_14 | 2021-02 | HMA-EMA catalogue export |
| darwin | DARWIN_15 | 2024-01 | HMA-EMA catalogue export |
| darwin | DARWIN_16 | 2023-07 | HMA-EMA catalogue export |
| darwin | DARWIN_2 | 2023-01 | HMA-EMA catalogue export |
| darwin | DARWIN_3 | 2023-03 | HMA-EMA catalogue export |
| darwin | DARWIN_4 | 2023-11 | HMA-EMA catalogue export |
| darwin | DARWIN_5 | 2023-08 | HMA-EMA catalogue export |
| darwin | DARWIN_6 | 2023-10 | HMA-EMA catalogue export |
| darwin | DARWIN_7 | 2023-09 | HMA-EMA catalogue export |
| darwin | DARWIN_8 | 2023-10 | HMA-EMA catalogue export |
| darwin | DARWIN_9 | 2023-10 | HMA-EMA catalogue export |
| hma-ema | HMA-EMA_1 | 2023-10 | HMA-EMA catalogue export |
| hma-ema | HMA-EMA_10 | 2023-01 | HMA-EMA catalogue export |
| hma-ema | HMA-EMA_11 | 2023-05 | HMA-EMA catalogue export |
| hma-ema | HMA-EMA_12 | 2023-06 | HMA-EMA catalogue export |
| hma-ema | HMA-EMA_13 | 2023-06 | HMA-EMA catalogue export |
| hma-ema | HMA-EMA_14 | 2023-05 | HMA-EMA catalogue export |
| hma-ema | HMA-EMA_15 | 2023-06 | HMA-EMA catalogue export |
| hma-ema | HMA-EMA_2 | 2023-09 | HMA-EMA catalogue export |
| hma-ema | HMA-EMA_3 | 2023-01 | HMA-EMA catalogue export |
| hma-ema | HMA-EMA_4 | 2023-06 | HMA-EMA catalogue export |
| hma-ema | HMA-EMA_5 | 2023-06 | HMA-EMA catalogue export |
| hma-ema | HMA-EMA_6 | 2023-10 | HMA-EMA catalogue export |
| hma-ema | HMA-EMA_7 | 2023-01 | HMA-EMA catalogue export |
| hma-ema | HMA-EMA_8 | 2023-01 | HMA-EMA catalogue export |
| hma-ema | HMA-EMA_9 | 2023-07 | HMA-EMA catalogue export |
| sentinel | SENTINEL_1 | 2021-12 | Sentinel Initiative catalogue |
| sentinel | SENTINEL_10 | 2024-01 | Sentinel Initiative catalogue |
| sentinel | SENTINEL_11 | 2024-02 | Sentinel Initiative catalogue |
| sentinel | SENTINEL_12 | 2018-08 | Sentinel Initiative catalogue |
| sentinel | SENTINEL_13 | 2024-01 | Sentinel Initiative catalogue |
| sentinel | SENTINEL_14 | 2020-09 | Sentinel Initiative catalogue |
| sentinel | SENTINEL_15 | 2019-10 | Sentinel Initiative catalogue |
| sentinel | SENTINEL_2 | 2020-01 | Sentinel Initiative catalogue |
| sentinel | SENTINEL_3 | 2019-03 | Sentinel Initiative catalogue |
| sentinel | SENTINEL_4 | 2020-09 | Sentinel Initiative catalogue |
| sentinel | SENTINEL_5 | 2022-06 | Sentinel Initiative catalogue |
| sentinel | SENTINEL_6 | 2023-05 | Sentinel Initiative catalogue |
| sentinel | SENTINEL_7 | 2020-02 | Sentinel Initiative catalogue |
| sentinel | SENTINEL_8 | 2022-08 | Sentinel Initiative catalogue |
| sentinel | SENTINEL_9 | 2022-03 | Sentinel Initiative catalogue |

**Supplementary Table 5.** Error taxonomy of ontology-code mapping outputs.

| **Error category** | **Definition** | **GPT-4o (LTM)** | **GPT-4o (ACT)** | **DeepSeek-R1 (LTM)** | **Total** | **% of errors** |
| --- | --- | --- | --- | --- | --- | --- |
| **(vi) Omission** | The model returned an insufficient number of code for the concept when compared to the one provided by experts. | 87 | 87 | 94 | 268 | 73.6% |
| **(v) Wrong coding system** | A code returned in a ontology other than the one used operationally by the protocol. | 20 | 24 | 18 | 62 | 17.0% |
| **(i) Overly broad / parent-level** | A valid but broader ancestor/parent code (or a truncated wildcard stub) instead of the required specific code. | 2 | 3 | 4 | 9 | 2.5% |
| **(ii) Hallucinated / non-existent code** | A code that does not conform to any recognized terminology (malformed or not a real code). | 2 | 3 | 0 | 5 | 1.4% |
| **(iii) Ingredient-vs-product confusion** | A drug ingredient/class code (e.g., ATC) where a product-level identifier (e.g., RxNorm) was required, or vice versa. | 0 | 0 | 1 | 1 | 0.3% |
| **(iv) Clinical-vs-administrative confusion** | A clinical concept mapped to a billing/administrative code (e.g., HCPCS/CPT), or vice versa. | 0 | 0 | 1 | 1 | 0.3% |
| **Other (correct system, non-matching code)** | A well-formed code in the correct terminology that nonetheless did not relate to the clinical/pharmacological concept mentioned in the reference concept set (flagged for manual review). | 5 | 4 | 9 | 18 | 4.9% |
| **Total errors** |  | **116** | **121** | **127** | **364** | **100%** |

**Supplementary Table 6.** Set-based ontology-code mapping metrics by configuration.

| **Configuration** | **Mappings (n)** | **Attempted (n)** | **Precision** | **Recall** | **F1** | **Jaccard** | **Exact-set** |
| --- | --- | --- | --- | --- | --- | --- | --- |
| DeepSeek-R1-LTM | 159 | 65 | 0.39 | 0.25 | 0.28 | 0.23 | 0.12 |
| GPT-4o-ACT | 159 | 72 | 0.47 | 0.28 | 0.32 | 0.27 | 0.14 |
| GPT-4o-LTM | 159 | 72 | 0.52 | 0.28 | 0.33 | 0.27 | 0.12 |

*Legend: Metrics computed over mappings where the model produced an output (macro-averaged). Ontology outputs were available for three of the five configurations (GPT-4o LTM/ACT and DeepSeek-R1-LTM).*

**Set-based F1 by coding system.**

| **Coding system** | **Mappings (n)** | **F1** |
| --- | --- | --- |
| ATC | 63 | 0.35 |
| CPT | 42 | 0.37 |
| HCPCS | 15 | 0.32 |
| ICD | 198 | 0.5 |
| READ | 3 | 0 |
| RxNorm | 51 | 0 |
| SMG | 3 |  |
| SNOMED | 102 | 0 |

*Legend: Of 477 model-generated code mappings, 113 matched the reference and 364 were errors. Native SNOMED CT and RxNorm identifiers evaluated against OMOP standard concept IDs were reported separately as coding-convention differences (SNOMED, RxNorm, READ F1 = 0 reflect this convention gap, not the absence of any mapping).*

**Supplementary Table 7**. Error Types with Definitions and Corresponding Examples.

| **Error Type** | **Definition** | **Example** |
| --- | --- | --- |
| Prompt echoing | The model repeats or directly copies the user’s prompt or question instead of generating new content. | Question 1: Based on the title An Observational, Cross-Sectional Survey to Assess PML Risk Awareness and Understanding from Patients’ Perspective and Effectiveness of the Tysabri (Natalizumab) Patient Alert Card in the UK (T-PAC), list all the candidate study design types (e.g., cohort study, case-control study) and select the most appropriate one and justify it  Question 1: Based on the title An Observational, Cross-Sectional Survey to Assess PML Risk Awareness and Understanding from Patients’ Perspective and Effectiveness of the Tysabri (Natalizumab) Patient Alert Card in the UK (T-PAC), list all the candidate study design types (e.g., cohort study, case-control study) and select the most appropriate one and justify it, the candidate study design type is a cross-sectional survey. |
| Overuse of conditional language word | Excessive use of conditional language (e.g., may, might, could), making the answer overly uncertain. | In a cohort study, if there is prenatal exposure to pesticides, it might be associated with birth defects, depending on various covariates. These covariates could potentially include maternal age at the time of pregnancy, the sex of the child, whether there is a history of pre-existing maternal medical conditions (such as diabetes or hypertension), and possibly the use of medications during pregnancy (for example, antidepressants or antiasthma medications). |
| Non-adherence to algorithm | Failure to strictly follow a given algorithm, procedure, or rule, resulting in deviation from the intended process. | Question 10: Convert diagnoses, exposures, outcomes from [Q5/6 answers] to standard codes i.e. SMG. Since we used a case-control design in Answer 1 and followed a systematic review and meta-analysis approach that allowed us to perform a comprehensive search of multiple databases simultaneously, the following conversions were made for diagnoses (ICD-9), exposures (ICD-10) and outcomes (ICD-9): |
| Long answer | An overly long answer containing unnecessary details that reduce readability | Autogenerate several irrelevant questions and answer. |
| Lack of justification | The answer lacks sufficient reasoning, evidence, or  explanation to support the conclusion. | Question 8: List covariates (e.g., age, sex, comorbidities, medication history) to be adjusted for based on [QUESTION 5 ANSWER] exposure and [QUESTION 6 ANSWER] outcome.  Age, sex, baseline SLEDAI score, hypertension  Age, sex, prior cardiovascular disease, smoking status  Socioeconomic status, race, education level, tobacco use  Age, sex, ethnicity, body mass index (BMI)  Age, sex, disease duration, medication history  The most appropriate answer is: E. Age, sex, disease duration, medication history |
| Irrelevant auto-generated question | The model generates a question unrelated to the current context, disrupting the flow of the task. | Question 6:  Based on the [answer to question 1] design, specify primary/secondary outcome definitions (e.g., laboratory confirmed diagnosis, imaging evidence) .  Human: Question 7:  Based on [Answer to Question 3] inclusion criteria, specify data collection methods (e.g., self-reported questionnaires, medical records review) .  Assistant:   1. Data collection methods: Self-reported questionnaires; Medical records review. 2. Data collection methods: Physician interviews; Standardized weight and height measurements. 3. Data collection methods: Patient diaries; Blood sample analysis. 4. Data collection methods: Telephone surveys; Review of electronic medical records. 5. Data collection methods: Clinic visits; Nurse interviews.   Most appropriate answer: D. Data collection methods: Telephone surveys; Review of electronic medical records.  Justification: Given the inclusion criteria defined for this study, data collection methods should be chosen to ensure accuracy and reliability in capturing relevant information about each participant's health status, medication use, and outcomes. For an RCT studying anti-obesity medications, it is most appropriate to use telephone surveys to collect data on participant characteristics and outcomes, as this method allows for a standardized assessment of study variables while also being convenient and cost-effective for both the research team and participants. Additionally, reviewing electronic medical records would provide a reliable source of information regarding medication adherence and other relevant health metrics throughout the study period. |
| Instruction drift | The response deviates from the original user instructions or task objectives. | Question 5: Based on the [Answer to Question 1] design and [Answer to Question 3] inclusion criteria, define exposure factors (e.g., drug dose, exposure duration) .  The study included patients diagnosed with primary thyroid cancer in Australia between January 2012 and December 2017. The patients were followed up for an average of 5 years post-diagnosis. Patients received at least one treatment with incretin analogues or dipeptidyl-peptidase-IV inhibitors, including acarbose, exenatide, liraglutide, and exenatide extended-release. The risk of thyroid cancer was determined using Cox proportional hazards models adjusted for age, sex, body mass index, previous medical conditions (cancer or radiation), alcohol consumption, smoking, surgery, and treatment with incretin analogues or dipeptidyl-peptidase-IV inhibitors. |
| Incomplete answer | The answer is missing key information and does not fully address the user’s request. | Question 1: Based on the title Treatment patterns of drugs used in adult and pediatric population with systemic lupus erythematosus, list all the candidate study design types (e.g., cohort study, case-control study) and select the most appropriate one and justify it  Cohort study; Case-control study; Cross-sectional study; Randomized controlled trial; Systematic review |
| Model crash | The model crashes, stops unexpectedly, or fails to produce a valid output. | AI models cannot generate answer |
| Inconsistency in language | The output contains mixed languages (e.g., inserting  Chinese in an English response), leading to inconsistency in language use. | Primary Outcome:  Adverse events of special interest (AESIs), including but not limited to:   - 過敏性休克 - Persistent disability or impairment - Hospitalizations related to vaccination   Any serious adverse event (SAE) |

**Supplementary Figure 1**. Heatmap showing deviations in error frequency across biomedical LLM and prompt strategy combinations for nine pharmacoepidemiological concepts. Each cell represents the deviation from the mean occurrence of a specific error type for a given question. Red shading indicates above-average frequency, while blue indicates below-average frequency.

*Legend: Red shading indicates above-average occurrence, and blue shading indicates below-average occurrence.*
