# Supplementary material for: Employing General-Purpose and Biomedical Large Language Models with Advanced Prompt Engineering for Pharmacoepidemiologic Study Design
Source: Pharm Res. 2026 Sep 11;43(10):3701–15. doi: 10.1007/s11095-026-04190-5 (PMC13736468; doi:10.1007/s11095-026-04190-5)
Supplement: Supplementary file 2 [file 11095_2026_4190_MOESM2_ESM.docx]

**Appendix 1.** Pre-assessment screening of 42 model–prompt combinations.

| **model (screening label)** | **actual model** | **prompt** | **Available coherence measurements** | **Mean coherence** | **Available relevance measurements** | **Mean relevance** | **Mean combined (relevance and coherence)** | **Model retained** |
| --- | --- | --- | --- | --- | --- | --- | --- | --- |
| PMC-LLAMA | QuantFactory/Bio-Medical-Llama-3-8B-GGUF (Q4_K_M) | Synthetic | 27 | 3.37 | 27 | 3.52 | 3.44 |  |
| PMC-LLAMA | QuantFactory/Bio-Medical-Llama-3-8B-GGUF (Q4_K_M) | zero-shot | 27 | 2.96 | 27 | 2.85 | 2.9 |  |
| PMC-LLAMA | QuantFactory/Bio-Medical-Llama-3-8B-GGUF (Q4_K_M) | Active | 27 | 3.85 | 27 | 3.11 | 3.48 |  |
| PMC-LLAMA | QuantFactory/Bio-Medical-Llama-3-8B-GGUF (Q4_K_M) | Plan-and-Solve | 27 | 3.89 | 27 | 3.52 | 3.7 |  |
| **PMC-LLAMA** | **QuantFactory/Bio-Medical-Llama-3-8B-GGUF (Q4_K_M)** | **Least-to-Most** | **27** | **3.81** | **27** | **3.37** | **3.59** | **YES** |
| PMC-LLAMA | QuantFactory/Bio-Medical-Llama-3-8B-GGUF (Q4_K_M) | Tree-of-Thought | 27 | 3.81 | 27 | 3.3 | 3.56 |  |
| PMC-LLAMA | QuantFactory/Bio-Medical-Llama-3-8B-GGUF (Q4_K_M) | Decomposition | 27 | 1 | 27 | 1 | 1 |  |
| Plaban81 | Plaban81/gemma-medical_qa-Finetune | zero-shot | 27 | 3.22 | 27 | 2.74 | 2.98 |  |
| Plaban81 | Plaban81/gemma-medical_qa-Finetune | Synthetic | 27 | 3.63 | 27 | 2.78 | 3.2 |  |
| Plaban81 | Plaban81/gemma-medical_qa-Finetune | Active | 27 | 3.63 | 27 | 2.78 | 3.2 |  |
| Plaban81 | Plaban81/gemma-medical_qa-Finetune | Plan-and-Solve | 27 | 3.63 | 27 | 3.04 | 3.34 |  |
| Plaban81 | Plaban81/gemma-medical_qa-Finetune | Least-to-Most | 27 | 3.67 | 27 | 3.22 | 3.44 |  |
| Plaban81 | Plaban81/gemma-medical_qa-Finetune | Tree-of-Thought | 27 | 3.7 | 27 | 3.04 | 3.37 |  |
| Plaban81 | Plaban81/gemma-medical_qa-Finetune | Decomposition | 27 | 3.44 | 27 | 2.81 | 3.12 |  |
| mradermacher | mradermacher/DeepSeek-r1-Medical-Mini-GGUF | zero-shot | 0 |  | 0 |  |  |  |
| mradermacher | mradermacher/DeepSeek-r1-Medical-Mini-GGUF | Synthetic | 0 |  | 0 |  |  |  |
| mradermacher | mradermacher/DeepSeek-r1-Medical-Mini-GGUF | Active | 0 |  | 0 |  |  |  |
| mradermacher | mradermacher/DeepSeek-r1-Medical-Mini-GGUF | Least-to-Most | 0 |  | 0 |  |  |  |
| mradermacher | mradermacher/DeepSeek-r1-Medical-Mini-GGUF | Tree-of-Thought | 0 |  | 0 |  |  |  |
| mradermacher | mradermacher/DeepSeek-r1-Medical-Mini-GGUF | Decomposition | 0 |  | 0 |  |  |  |
| mradermacher | mradermacher/DeepSeek-r1-Medical-Mini-GGUF | Plan-and-Solve | 0 |  | 0 |  |  |  |
| Irathernotsay | Irathernotsay/qwen2-1.5B-medical_qa-Finetune | zero-shot | 27 | 3.59 | 27 | 2.7 | 3.14 |  |
| Irathernotsay | Irathernotsay/qwen2-1.5B-medical_qa-Finetune | Synthetic | 27 | 3.48 | 26 | 2.65 | 3.06 |  |
| Irathernotsay | Irathernotsay/qwen2-1.5B-medical_qa-Finetune | Active | 27 | 3.74 | 27 | 3 | 3.37 |  |
| Irathernotsay | Irathernotsay/qwen2-1.5B-medical_qa-Finetune | Plan-and-Solve | 27 | 3.74 | 27 | 3 | 3.37 |  |
| **Irathernotsay** | **Irathernotsay/qwen2-1.5B-medical_qa-Finetune** | **Least-to-Most** | **27** | **3.89** | **27** | **3.19** | **3.54** | **YES** |
| Irathernotsay | Irathernotsay/qwen2-1.5B-medical_qa-Finetune | Tree-of-Thought | 27 | 3.67 | 27 | 2.81 | 3.24 |  |
| Irathernotsay | Irathernotsay/qwen2-1.5B-medical_qa-Finetune | Decomposition | 27 | 3.48 | 27 | 2.74 | 3.11 |  |
| gpt-4o | GPT-4o (gpt-4o-2024-08-06) | zero-shot | 27 | 4.44 | 27 | 3.89 | 4.16 |  |
| gpt-4o | GPT-4o (gpt-4o-2024-08-06) | Synthetic | 27 | 4.33 | 27 | 3.89 | 4.11 |  |
| **gpt-4o** | **GPT-4o (gpt-4o-2024-08-06)** | **Active** | **30** | **4.3** | **30** | **3.93** | **4.12** | **YES** |
| gpt-4o | GPT-4o (gpt-4o-2024-08-06) | Plan-and-Solve | 30 | 4.2 | 30 | 3.67 | 3.94 |  |
| **gpt-4o** | **GPT-4o (gpt-4o-2024-08-06)** | **Least-to-Most** | **30** | **4.4** | **30** | **3.87** | **4.14** | **YES** |
| gpt-4o | GPT-4o (gpt-4o-2024-08-06) | Tree-of-Thought | 30 | 4.1 | 30 | 3.53 | 3.81 |  |
| gpt-4o | GPT-4o (gpt-4o-2024-08-06) | Decomposition | 30 | 3.9 | 30 | 3.6 | 3.75 |  |
| deepseek | DeepSeek-R1 | zero-shot | 0 |  | 0 |  |  |  |
| deepseek | DeepSeek-R1 | Synthetic | 0 |  | 0 |  |  |  |
| deepseek | DeepSeek-R1 | Active | 0 |  | 0 |  |  |  |
| **deepseek** | **DeepSeek-R1** | **Least-to-Most** | **0** |  | **0** |  |  | **YES** |
| deepseek | DeepSeek-R1 | Plan-and-Solve | 0 |  | 0 |  |  |  |
| deepseek | DeepSeek-R1 | Tree-of-Thought | 0 |  | 0 |  |  |  |
| deepseek | DeepSeek-R1 | Decomposition | 0 |  | 0 |  |  |  |

*Legend / decision rule*: Feasibility-driven prioritization, not a strict numeric threshold. Retained configurations (5, shaded) correspond to the five evaluated model–prompt configurations. "actual model" gives the Hugging Face checkpoint / model behind each screening label. PMC-LLAMA (screening label) = QuantFactory/Bio-Medical-Llama-3-8B-GGUF (Q4_K_M) — the retained biomedical Llama; Irathernotsay = qwen2-1.5B-medical (retained); gpt-4o = GPT-4o gpt-4o-2024-08-06; deepseek = DeepSeek-R1 (retained a priori as second general-purpose reasoning comparator, not numerically screened); mradermacher = DeepSeek-r1-Medical-Mini-GGUF (no scorable output, 0/7); Plaban81 = gemma-medical_qa-Finetune. Least-to-Most was adopted as the common cross-model prompt (most consistent across families); GPT-4o additionally retained under Active for the model-controlled prompt comparison; the two highest-scoring biomedical models (Bio-Medical-Llama-3-8B 3.59, Qwen2-1.5B-medical 3.54) were carried forward at Least-to-Most.
